# DJ-1 inhibits microglial activation and protects dopaminergic neurons in vitro and in vivo through interacting with microglial p65

**DOI:** 10.1038/s41419-021-04002-1

**Published:** 2021-07-17

**Authors:** Zixuan Lin, Chen Chen, Dongqin Yang, Jianqing Ding, Guanghui Wang, Haigang Ren

**Affiliations:** 1 Jiangsu Key Laboratory of Translational Research and Therapy for Neuropsychiatric disorders & Department of Pharmacology, College of Pharmaceutical Sciences, Soochow University, 199 Ren’ai Road, Suzhou, Jiangsu 215123 China; 2 Department of Neurology & Institute of Neurology, Ruijin Hospital affiliated to Shanghai Jiao Tong University School of Medicine, Shanghai, 200025 China

**Keywords:** Cell death in the nervous system, Parkinson's disease

## Abstract

Parkinson’s disease (PD), one of the most common neurodegenerative disorders, is characterized by progressive neurodegeneration of dopaminergic (DA) neurons in the substantia nigra pars compacta (SNpc). DJ-1 acts essential roles in neuronal protection and anti-neuroinflammatory response, and its loss of function is tightly associated with a familial recessive form of PD. However, the molecular mechanism of DJ-1 involved in neuroinflammation is largely unclear. Here, we found that wild-type DJ-1, rather than the pathogenic L166P mutant DJ-1, directly binds to the subunit p65 of nuclear factor-κB (NF-κB) in the cytoplasm, and loss of DJ-1 promotes p65 nuclear translocation by facilitating the dissociation between p65 and NF-κB inhibitor α (IκBα). *DJ-1* knockout (*DJ-1*^−/−^) mice exhibit more microglial activation compared with wild-type littermate controls, especially in response to lipopolysaccharide (LPS) treatment. In cellular models, knockdown of DJ-1 significantly upregulates the gene expression and increases the release of LPS-treated inflammatory cytokines in primary microglia and BV2 cells. Furthermore, *DJ-1* deficiency in microglia significantly enhances the neuronal toxicity in response to LPS stimulus. In addition, pharmacological blockage of NF-κB nuclear translocation by SN-50 prevents microglial activation and alleviates the damage of DA neurons induced by microglial *DJ-1* deficiency in vivo and in vitro. Thus, our data illustrate a novel mechanism by which DJ-1 facilitates the interaction between IκBα and p65 by binding to p65 in microglia, and thus repressing microglial activation and exhibiting the protection of DA neurons from neuroinflammation-mediated injury in PD.

## Introduction

Parkinson’s disease (PD) is one of the most common neurodegenerative diseases, with a prevalence of more than 1% in the population over 65 years old, and up to ~5% over age 85 [1, 2]. The typical pathological feature of PD is the progressive loss of dopaminergic (DA) neurons, that is selectively detected in the substantia nigra pars compacta (SNpc) [3]. Genetic factors, aging, and neurotoxins contribute to PD pathogenesis [4]. Thus far, more than 20 genes including *DJ-1*/*PARK7* have been identified to be related to multiple forms of familial PD [5]. A great deal of evidence indicates that neuroinflammation-mediated DA neurotoxicity acts a vital role in the pathogenesis of both familial and sporadic forms of PD [6, 7].

Microglia are macrophages that reside in the central nervous system (CNS), playing key roles in brain immunity and mediate neuroinflammation in response to neuronal injury or dysfunction [8]. Overactivation of microglia leads to excess production of pro-inflammatory factors including inducible nitric oxide synthase (iNOS), cyclooxygenase-2 (COX-2), tumor necrosis factor-α (TNFα), prostaglandin E2 (PGE2), interleukin-6 (IL-6), and nitric oxide (NO) [9], which lead to DA neuronal death in PD [10]. A PD-associated protein, DJ-1 homozygous deletion or point mutations including L166P are associated with early-onset autosomal recessive forms of PD [11, 12]. Moreover, altered levels of DJ-1 are also found in sporadic PD patients [13–16]. DJ-1 protein is abundantly expressed in both neurons and glial cells in the CNS, and is mainly distributed in the cytosol and partially in the nucleus and mitochondria [11, 17–19]. It has been demonstrated that DJ-1 protects DA neurons through its multifunctional roles in anti-oxidative ability, transcriptional regulation, mitochondrial function regulation, and signal transduction in neurons [20]. Recent reports also point out that DJ-1 acts a vital role in the neuroinflammatory response, and the downregulation of DJ-1 augments neuroinflammation in glial cells [21–24]. However, the role of microglial DJ-1 in vivo, as well as the potential molecular mechanisms of DJ-1 in microglia are largely unclear.

Here, we reveal a novel mechanism by which DJ-1 directly binds to p65 in microglial cytoplasm to block neuroinflammation. DJ-1 deficiency facilitates the dissociation between p65 and IκBα, leading to p65 nuclear translocation and increases nuclear factor-κB (NF-κB) transcriptional activity. *DJ-1*^−/−^ mice exhibit more microglial activation than wild-type in response to LPS treatment. Moreover, DJ-1 deficiency significantly increases the production of inflammatory factors in microglia and results in DA neuronal loss in response to LPS stimulus. In addition, NF-κB inhibitors block microglial activation as well as neuronal cell death induced by DJ-1 deficiency in vivo and in vitro. Thus, our data illustrate a novel mechanism consisting of DJ-1 inhibiting neuroinflammation by facilitating the interaction between IκBα and p65 in microglia.

## Results

### DJ-1 deficiency leads to microglial activation and DA neuron loss

**Fig. 1:**
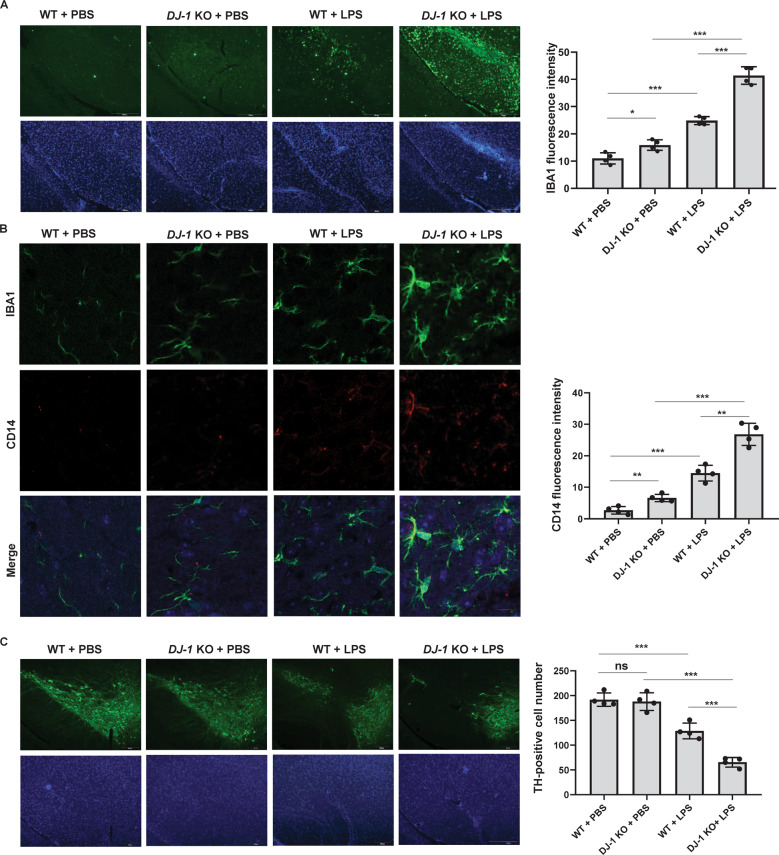
DJ-1 deficiency leads to microglia activation and DA neuron loss in vivo. **A**–**C** *DJ-1*^−/−^ and the littermate wild-type controls were microinfused with PBS or LPS into the SN, and 14 days after injection, immunohistochemical staining was conducted. **A** Slices were stained with anti-IBA1 antibodies. Scale bar, 200 µm. The fluorescence intensity of IBA1 staining was quantified. *n* = 4. **B** Slices were co-stained with anti-CD14 and anti-IBA1 antibodies. Scale bar, 20 µm. The fluorescence intensity of CD14 staining was quantified. *n* = 4. **C** Slices were stained with anti-TH antibodies. Scale bar, 200 µm. TH-positive neuron numbers in the SN of each slice were quantified. *n* = 4.

To examine the roles of DJ-1 on microglia in vivo, we first examined the microglia activation in the substantia nigra (SN) in *DJ-1*^−/−^ mice and the littermate wild-type controls, with or without LPS treatment. As shown in Fig. 1A, *DJ-1*^−/−^ mice exhibited a greater amount of IBA1 staining than the littermate wild-type controls, especially in response to LPS treatment. We then examined the expression of CD14, a marker of the pro-inflammatory phenotype of microglia, to access whether the increased microglia exhibited pro-inflammatory properties. A greater amount of CD14 expression was detected in *DJ-1*^−/−^ mice than that in the littermate wild-type controls. In addition, most of the IBA1-positive microglia were co-labeled with CD14 staining in *DJ-1*^−/−^ mice under LPS treatment (Fig. 1B). Although the number of DA neurons labelled with tyrosine hydroxylase (TH) in the SN of *DJ-1*^−/−^ mice was not significantly different from that of wild-type controls, LPS stimulation significantly reduced the number of DA neurons in *DJ-1*^−/−^ mice compared to wild-type controls (Fig. 1C).

**Fig. 2:**
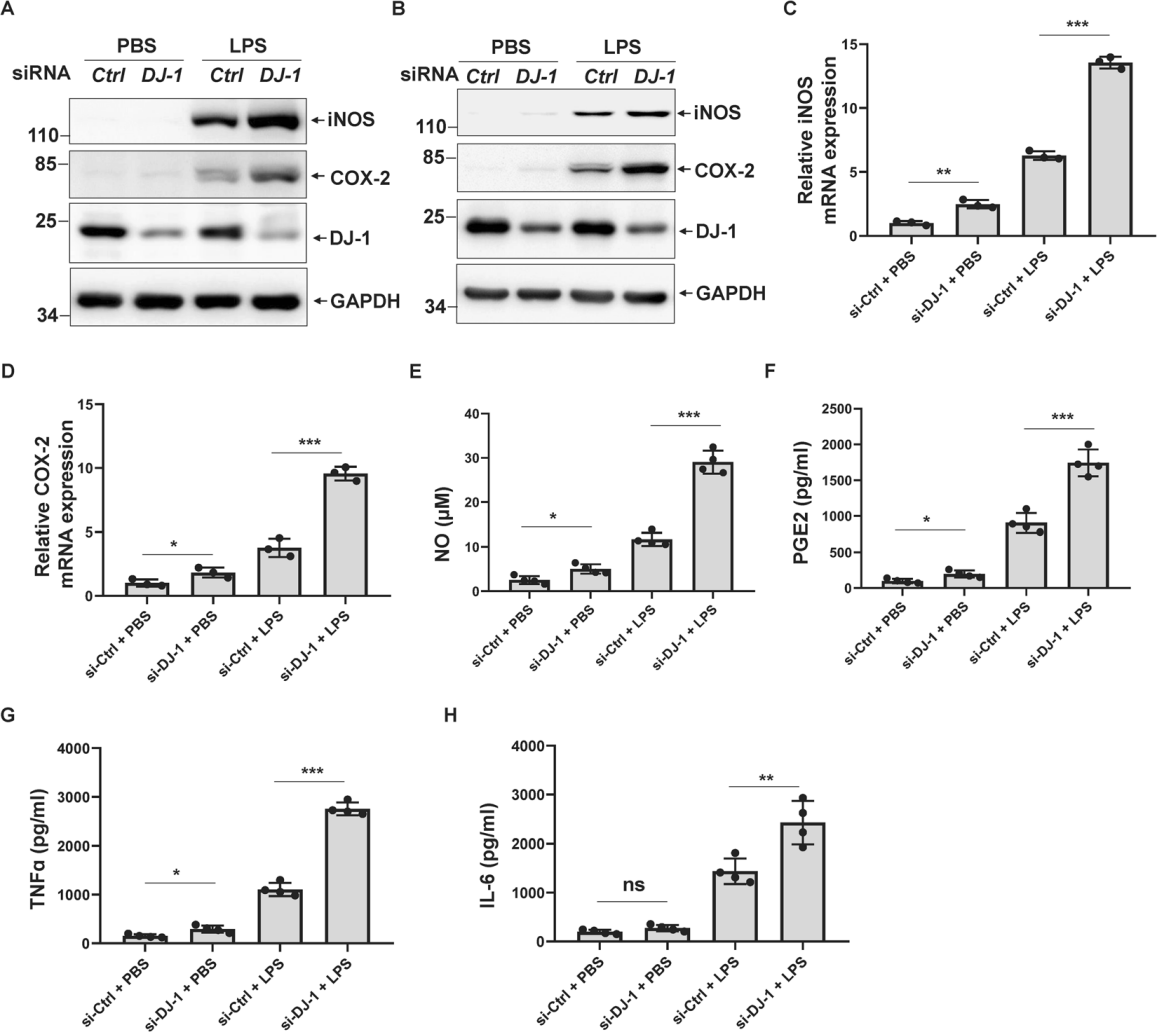
DJ-1 deficiency results in microglia activation in vitro. **A**, **B** si-*Ctrl* or si-*DJ-1* was transfected into primary microglia (**A**) or BV2 cells (**B**) for 48 h. The cells were then treated with PBS or LPS (100 ng/ml) for 24 h. The cell lysates were analyzed by immunoblotting using the indicated antibodies. **C**, **D** si-*Ctrl* or si-*DJ-1* was transfected into BV2 cells for 72 h. The cells were then treated with PBS or LPS (100 ng/ml) for 6 h and then were subjected to qRT-PCR to measure **C** *iNOS* or **D** *COX-2* mRNA levels. *n* = 3. **E**–**H** si-*Ctrl* or si-*DJ-1* was transfected into BV2 cells for 48 h. The cells were then treated with PBS or LPS (100 ng/ml) for 24 h. The concentration of **E** NO, **F** PGE2, **G** TNFα, or **H** IL-6 in the cultured medium was measured. *n* = 4.

To explore the mechanism by which DJ-1 deficiency causes microglia activation, we performed *DJ-1* siRNA experiments in mouse primary microglia and BV2 microglial cells. We first examined the expression of COX-2 and iNOS, two major inflammatory mediators. Although knockdown of DJ-1 alone induced a slight increase in COX-2 and iNOS expression levels both in primary microglia and BV2 cells (Fig. 2A, B), DJ-1 silencing dramatically increased COX-2 and iNOS levels compared with those in the control in response to LPS stimulation (Fig. 2A, B). In addition, DJ-1 knockdown significantly increased mRNA levels of *COX-2* and *iNOS*, especially in response to LPS treatment (Fig. 2C, D).

Next, we explored the effects of silencing *DJ-1* on the release of inflammatory cytokines using BV2 cells. Inflammatory cytokines NO and PGE2 are key downstream products of iNOS and COX-2, respectively [25, 26]. DJ-1 knockdown alone slightly increased NO and PGE2 release into the cultured media, whereas DJ-1 deficiency dramatically increased the release of NO and PGE2 in response to LPS stimulation (Fig. 2E, F). In addition, the knockdown of DJ-1 also increased the production of TNFα and IL-6 under LPS treatment (Fig. 2G, H). These data suggest that the loss of DJ-1 facilitates microglial activation and intrinsically increases the production of various pro-inflammatory cytokines, especially in response to LPS stimulation.

### DJ-1 but not its pathogenic L166P mutant represses NF-κB transcriptional activity

**Fig. 3:**
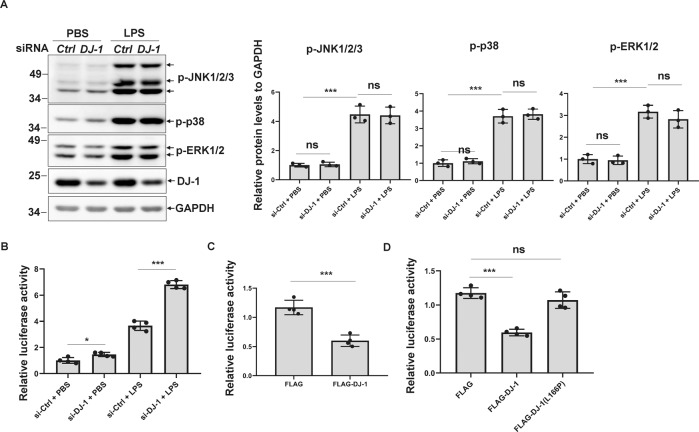
DJ-1 rather than L166P mutant represses NF-κB transcriptional activity. **A** si-*Ctrl* or si-*DJ-1* was transfected into BV2 cells for 72 h. Then the cells were treated with PBS or LPS (100 ng/ml) for 15 min. The cell lysates were analyzed by immunoblotting using the indicated antibodies. The bar graph shows the relative band intensity of p-JNK1/2/3, p-p38, or p-ERK1/2 to that of GAPDH. *n* = 3. **B** BV2 cells harboring lentiviral NF-κB-luc were transfected with si-*Ctrl* or si-*DJ-1*. After 48 h, the cells were treated with PBS or LPS (100 ng/ml) for 24 h and then subjected to a luciferase reporter gene assay. *n* = 4. **C** HEK293 cells were transiently transfected with pNF-κB-luc and *Renilla* along with FLAG or FLAG-DJ-1. After 48 h, the cells were subjected to a luciferase reporter gene assay. One-way ANOVA, *n* = 4. **D** HEK293 cells were transiently transfected with pNF-κB-luc and *Renilla* along with FLAG, FLAG-DJ-1, or FLAG-DJ-1(L166P). After 48 h, the cells were subjected to a luciferase reporter gene assay. One-way ANOVA, *n* = 4.

Mitogen-activated protein kinases (MAPKs) including extracellular signal-regulated kinase (ERK), Jun N-terminal kinase (JNK), and p38 are important in the inflammatory response [27], and DJ-1 has been reported to influence their activity in some types of cells such as cancer cells, neurons, or astrocytes [28–32]. Thus, we first tested whether DJ-1 influenced JNK, p38, or ERK1/2 activity in microglial cells. Although LPS treatment immediately activated JNK, p38, and ERK1/2 phosphorylation, DJ-1 silencing had no significant effect on JNK, p38, or ERK activation, no matter whether BV2 cells were treated with LPS or not (Fig. 3A), suggesting that the effect of DJ-1 on microglial activation is not mediated by the MAPK pathway in microglia.

The expression levels of inflammatory factors are mainly regulated by the transcription factor NF-κB [25, 26], and thus, we next examined whether DJ-1 regulates NF-κB activity. In BV2 cells that stably express Cignal lentiviral NF-κB-luciferase, knockdown of DJ-1 alone significantly increased the NF-κB transcription activity (Fig. 3B), and this activation effect was further augmented under LPS stimulation (Fig. 3B).

We next tested the effect of DJ-1 overexpression on NF-κB transcriptional activity. Because the transfection efficiency of overexpression in BV2 cells is very low, we alternatively used HEK293 cells to perform the reporter gene assay. FLAG-DJ-1 overexpression but not the control plasmid significantly repressed NF-κB activity (Fig. 3C). However, the pathogenic DJ-1(L166P) lost the ability to repress NF-κB activity (Fig. 3D). These data indicate that wild-type DJ-1 but not the pathogenic DJ-1(L166P) regulates microglia-mediated neuroinflammation through the NF-κB signaling pathway.

### DJ-1 but not the pathogenic L166P mutant binds to the NF-κB subunit p65

**Fig. 4:**
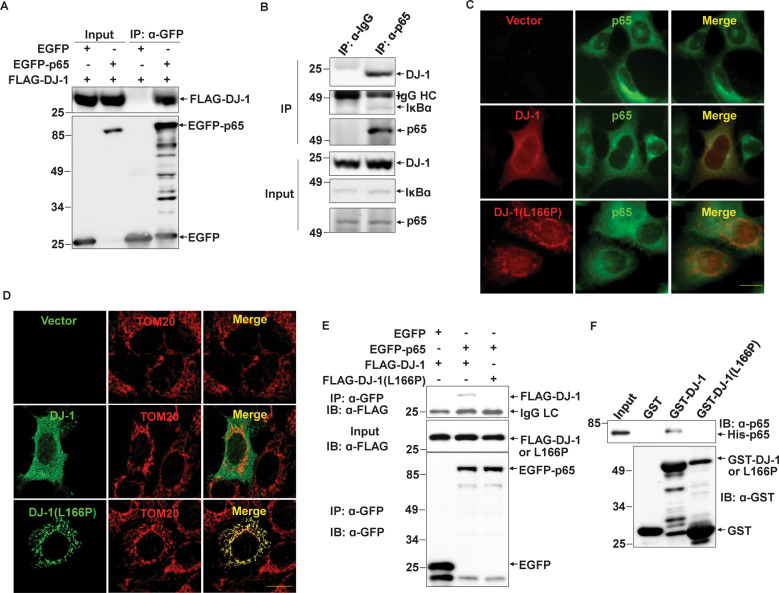
DJ-1 rather than L166P mutant binds to the p65. **A** The cell lysate supernatants of HEK293 cells transiently transfected with FLAG-DJ-1 along with EGFP or EGFP-p65 were immunoprecipitated using an anti-GFP antibody. **B** The supernatants of the BV2 cell lysates were immunoprecipitated using anti-p65 antibodies or normal rabbit IgG. **C** HEK293 cells transiently transfected with FLAG, FLAG-DJ-1, or FLAG-DJ-1(L166P) were subjected to immunocytochemistry using anti-p65 (green) and anti-FLAG (red) antibodies. Scale bar, 10 µm. **D** HEK293 cells transiently transfected with FLAG, FLAG-DJ-1, or FLAG-DJ-1(L166P) were subjected to immunocytochemistry using anti-TOM20 (red) and anti-FLAG (green) antibodies with a confocal microscope. Scale bar, 10 µm. **E** The cell lysate supernatants of HEK293 cells transiently transfected with FLAG-DJ-1 or FLAG-DJ-1(L166P) along with EGFP or EGFP-p65 were subjected to immunoprecipitation with anti-GFP antibodies. **F** Recombinant GST, GST-DJ-1, or GST-DJ-1(L166P) and His-p65 were subjected to GST-pull-down assay.

Many studies have shown that DJ-1 can influence transcriptional factors through direct or indirect interactions [33]. We performed co-immunoprecipitation experiments to examine whether DJ-1 interacts with p65, an NF-κB subunit. In HEK293 cells, Flag-DJ-1 could be pulled down by EGFP-p65 rather than EGFP using an anti-GFP antibody (Fig. 4A). Further, endogenous DJ-1 could be co-immunoprecipitated with p65 using anti-p65 antibodies but not control IgG in BV2 cells (Fig. 4B).

We next detected the subcellular distribution of p65 and DJ-1 in cells. DJ-1 and p65 exhibited co-localization in the cytoplasm but not in the nucleus (Fig. 4C). In addition, DJ-1(L166P) exhibited much more mitochondrial localization and less co-localization with p65 in the cytoplasm (Fig. 4C, D). The co-immunoprecipitation experiment also indicated that wild-type DJ-1 but not L166P mutant interacted with p65 in cells (Fig. 4E).

To further identify whether there is a direct interaction between DJ-1 and p65, a GST-pull-down assay using purified recombinant proteins was performed. His-p65 could be directly pulled down by GST-DJ-1 rather than GST or GST-DJ-1(L166P) (Fig. 4F), indicating that wild-type DJ-1 binds to p65 and represses the transcription activity of NF-κB, while the pathogenic L166P mutant DJ-1 loses this ability.

### DJ-1 represses p65 nuclear translocation in response to LPS treatment

**Fig. 5:**
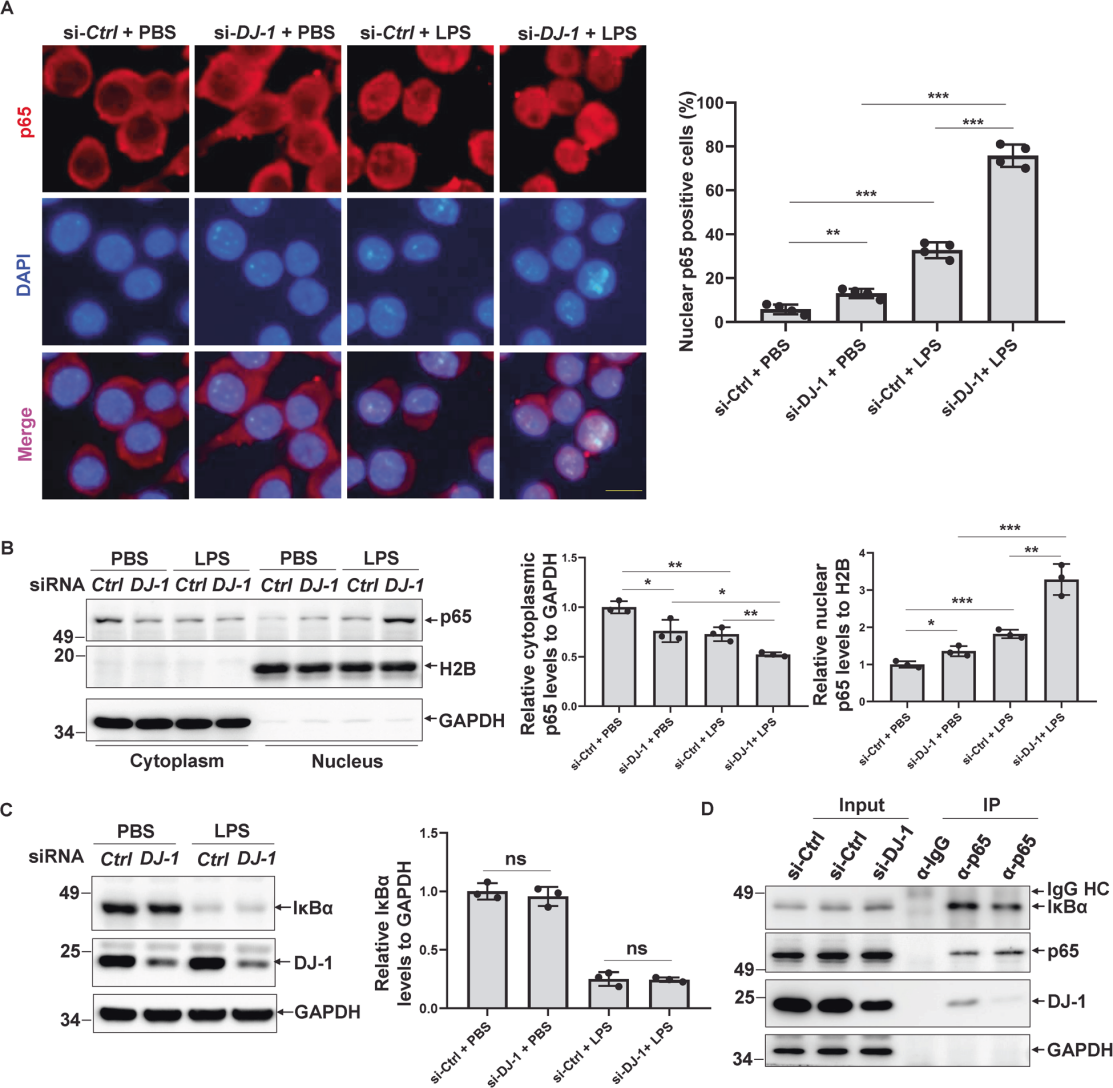
DJ-1 represses p65 nuclear translocation. **A**–**C** BV2 cells were transfected with si-*Ctrl* or si-*DJ-1* for 72 h and then treated with PBS or LPS (100 ng/ml) for 15 min. **A** The cells were stained with anti-p65 antibody and DAPI. Scale bar, 10 µm. And the percentage of cells with nuclear p65 distribution was quantified. *n* = 4. **B** The cells were then subjected to a subcellular fractionation assay. The relative band intensity of cytoplasmic or nuclear p65 to that of GAPDH or H2B was quantified, respectively. *n* = 3. **C** Cell lysates were then subjected to immunoblotting. The relative band intensity of IκBα to that of GAPDH was quantified. *n* = 3. **D** The supernatants of the BV2 cells transfected with si-*Ctrl* or si-*DJ-1* for 72 h were immunoprecipitated using anti-p65 antibodies or normal rabbit IgG.

We found that DJ-1 interacts with p65 in the cytoplasm and inhibits NF-κB transcription activity (Figs. 3B–D and 4A–E), and p65 nuclear translocation is necessary for NF-κB transcription activity [26]. Therefore, we next examined whether DJ-1 had an effect on p65 nuclear translocation. Knockdown of DJ-1 induced a slight decrease in cytosolic p65 and a slight increase in nuclear p65 (Fig. 5A, B), whereas DJ-1 silencing resulted in a dramatic nuclear translocation of p65 in LPS-treated BV2 cells (Fig. 5A, B).

NF-κB inhibitor α (IκBα) is the major partner that interacts with and sequesters p65 in the cytoplasm, and thus inhibits NF-κB transcription activity, and in response to LPS stimulation, it is phosphorylated by IKK (IκB kinase) and rapidly degraded by the ubiquitin-proteasome system [25, 26]. Therefore, we examined whether DJ-1 deletion influences IκBα protein levels or the interaction between p65 and IκBα. However, *DJ-1* deletion did not affect IκBα protein levels with or without LPS treatment (Fig. 5C). Interestingly, the knockdown of DJ-1 reduced the interaction between IκBα and p65 in BV2 cells (Fig. 5D). These results indicate that DJ-1 facilitates the interaction between p65 and IκBα by binding to p65, and loss of DJ-1 promotes p65 nuclear translocation and thus activates NF-κB transcription activity.

### DJ-1 deficiency increases NF-κB-dependent microglial neurotoxicity

**Fig. 6:**
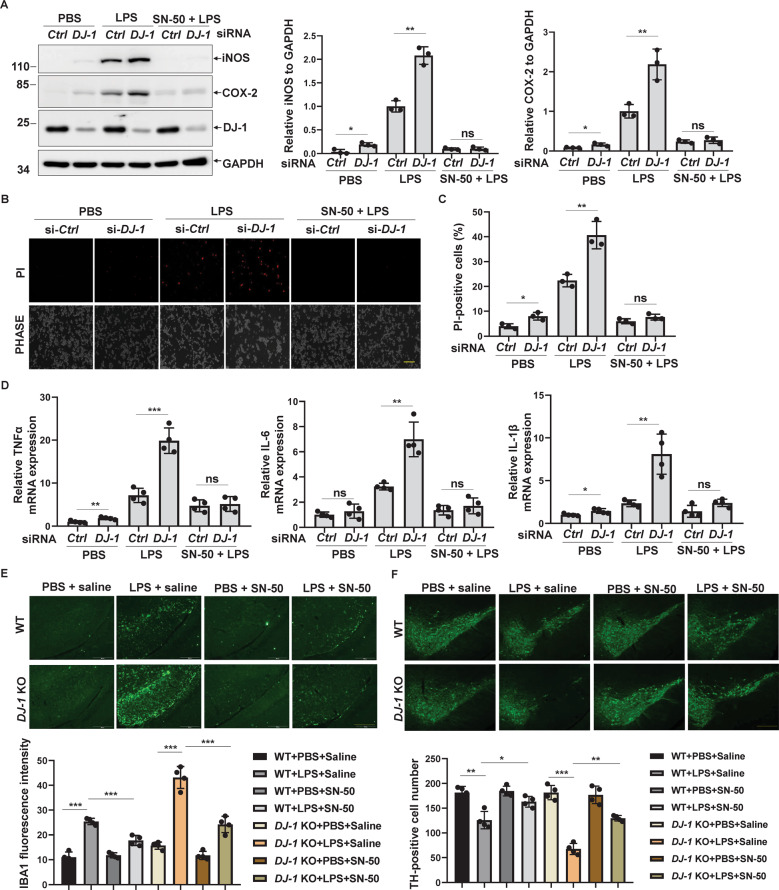
DJ-1 deficiency increases NF-κB-dependent microglial neurotoxicity. **A** BV2 cells transfected with si-*Ctrl* or si-*DJ-1* for 48 h were treated with PBS or LPS (100 ng/ml) for 24 h followed by pretreated with SN-50 (20 μM) for 1 h as indicated. After treatment, cell lysates were subjected to immunoblotting. The relative band intensity of iNOS or COX-2 to that of GAPDH was quantified. *n* = 3. **B** N2a cells cultured in conditioned media collected from BV2 cells with the indicated treatment for 24 h were then stained with PI and visualized with an inverted microscope. Scale bar, 50 µm. **C** The percentage of PI-positive cells in (**B**) was analyzed. *n* = 3. **D** Primary microglia transfected with si-*Ctrl* or si-*DJ-1* for 48 h were treated with PBS or LPS (100 ng/ml) for 6 h followed by pretreated with SN-50 (20 μM) for 1 h as indicated, then the samples were subjected to qRT-PCR. ^*^P < 0.05, *n* = 4. **E**, **F** *DJ-1*^−/−^ and the littermate wild-type controls were microinfused with PBS or LPS for 14 days followed by saline or SN-50 into the SN as indicated, and then the immunohistochemical staining was conducted using (**E**) anti-IBA1 antibody or (**F**) anti-TH antibody. The fluorescence intensity of IBA1 (**E**) and the number of TH-positive neurons (**F**) in SN of each slice were quantified. *n* = 4.

To further confirm whether microglia activation by DJ-1 deficiency is NF-κB dependent, we examined the inflammatory response in *DJ-1*-deficient BV2 cells with SN-50, a specific NF-κB inhibitor that directly inhibits NF-κB nuclear transport [34]. SN-50 completely blocked iNOS and COX-2 expression induced by *DJ-1* knockdown under LPS stimulation (Fig. 6A). The secretion of inflammatory cytokines by activated microglia is considered to be toxic to neuronal cells [35]. To determine whether loss of DJ-1 in microglia induces neurotoxicity, we performed propidium iodide (PI) fluorescence staining to determine the toxic effects of loss of microglial DJ-1 on N2a cells. Conditioned media from *DJ-1*-deficient BV2 cells slightly induced N2a cell death (Fig. 6B, C). Furthermore, conditioned media collected from *DJ-1*-knockdown BV2 cells in combination with LPS treatment exhibited a greater amount of cell death as compared to that from control siRNA-knockdown BV2 cells (Fig. 6B, C). Interestingly, SN-50 significantly inhibited neurotoxicity caused by loss of microglial DJ-1 combined with LPS stimulation (Fig. 6B, C). We also examined whether SN-50 inhibits the augmentation of DJ-1 deficiency-induced TNFα, IL-1β, and IL-6 expression in primary microglia in response to LPS stimulation. As shown in Fig. 6D, SN-50 significantly inhibited the mRNA levels of TNFα, IL-1β, and IL-6 induced by the knockdown of DJ-1 under LPS treatment.

We next wondered whether inhibiting the nuclear translocation of NF-κB blocks microglial activation and alleviates neurotoxicity by loss of microglial DJ-1 in vivo. Stereotactic injection of SN-50 into the SN dramatically inhibited the activation of microglia in *DJ-1*^−/−^ mice in response to LPS stimulation (Fig. 6E). SN-50 did not affect the number of DA neurons in both wild-type and the littermate *DJ-1*^−/−^ mice without LPS treatment (Fig. 6F). Whereas, SN-50 treatment dramatically alleviated the loss of DA neurons by LPS in *DJ-1*^−/−^ mice (Fig. 6F). Thus, these data revealed that lack of microglial DJ-1 results in an exaggerated inflammatory response through the promotion of NF-κB nuclear translocation, which increases DA neurotoxicity, and inhibition of NF-κB nuclear transport is a potential target for alleviating the damage to DA neurons in PD.

## Discussion

Microglia play vital roles in CNS hemostasis and are sensitive to activation in response to signals derived from dysfunctional neurons or neurotoxins. Activated microglia could produce and release pro-inflammatory factors such as TNFα, IL-1α/β, and IL-6, leading to damaged DA neurons in the SN [36]. Since it was discovered in 1988 that a large number of activated human leukocyte antigen (HLA)-positive microglia are present in the SNpc of PD patients [37], there has been a dramatically increased focus on the microglia in PD in recent years. Microglia-mediated neuroinflammation is an essential and common pathogenic factor in the early stage of PD and is extensively regarded as a therapeutic target of PD intervention. In addition to the direct damage to DA neurons, microglia-mediated neuroinflammation also induces astrocytes to adopt a neurotoxic function [38].

Recently, it has been reported that the PD-associated genetic factor DJ-1 controls important functions in astrocytes and microglia [21–24]. However, the role of DJ-1 in microglia in vivo and the potential molecular mechanisms of DJ-1 involves in microglial regulation are largely unclear. In the present study, we describe the functions and mechanisms of microglial DJ-1, a PD-related protein in neuroinflammatory regulation. Wild-type DJ-1 rather than its pathogenic mutant L166P blocks activation of microglia via the NF-κB signaling pathway and protects DA neurons from inflammatory damage in vitro and in vivo. In our observation, wild-type DJ-1 binds to and sequesters p65 in the cytoplasm, and thus repressing NF-κB hyperactivation, especially under LPS stimulation. Therefore, lacking *DJ-1* in mice, primary microglia and cultured BV2 cells causes a great increase in the pro-inflammatory phenotype of microglia compared with the controls, especially in response to LPS treatment (Figs. 1B and 2). In addition, it has been reported that DJ-1 has a great antioxidant function and reactive oxygen species (ROS) scavenging capacity [20, 33]. Interestingly, ROS are crucial for microglial polarization regulation and excessive ROS promote the pro-inflammatory phenotype of microglia [39]. So, we speculated that microglial activation induced by loss of DJ-1 may also be related to intracellular oxidative stress.

Two major transcription factors, NF-κB and activator protein 1 (AP-1) are both involved in microglial activation [40, 41]. MAPKs including ERK, JNK, and p38 are required for AP-1 activation to induce the expression of inflammatory factors [42, 43]. Although DJ-1 has been reported to affect the activity of these kinases in other types of cells [28–32], loss of DJ-1 did not significantly affect their activation in BV2 microglia with or without LPS treatment (Fig. 3A), suggesting that DJ-1 regulation of the inflammatory response is independent of the AP-1 pathway in microglia. In this study, we found that overexpression of DJ-1 inhibits NF-κB activity, and knockdown of DJ-1 activates NF-κB activity (Fig. 3B–D). In unstimulated cells, IκB binds to the p65/p50 heterodimer of NF-κB and sequesters NF-κB in the cytosol. Upon stimulation by TNFα, LPS, and ROS, for example, upon LPS recognition, Toll-like receptor 4 (TLR4) undergoes oligomerization, activates its downstream signaling pathway including the IKK complex. Then IκBα is phosphorylated by IKK and subsequently degraded by the ubiquitin-proteasome system, thus leading to a release in NF-κB that is transported into the nucleus and subsequently transactivates its target gene expression such as the expression of iNOS, COX-2, TNFα, IL-1, or IL-6 [25, 26].

Here, we found that wild-type DJ-1 rather than its pathogenic mutant L166P directly binds to the p65 subunit and facilitates the interaction between p65 and IκBα in the cytoplasm, as a DJ-1 deficiency leads to a reduction in their interaction (Fig. 5D). Loss of DJ-1 exacerbates the dissociation of p65 and IκBα, and promotes NF-κB nuclear localization, especially in response to inflammatory stimulation (Fig. 5A, B). Unlike wild-type DJ-1, which is distributed in the cytoplasm, binds to p65 and inhibits NF-κB activity, the L166P mutant DJ-1 translocates to mitochondria and cannot bind to p65 (Fig. 4C–E). It is also possible that the translocation of the L166P mutant DJ-1 to the mitochondria may affect mitochondrial function and promote the activation of microglia by binding to several specific mitochondrial proteins or affecting mitochondrial metabolism, which needs further investigation. Thus, these alterations make microglia harboring L166P mutant DJ-1 more sensitive to inflammatory activators such as LPS, and augments the inflammatory responses, thus aggravating the inflammatory damage to DA neurons. Interestingly, the NF-κB nuclear transport inhibitor SN-50 has a dominant anti-inflammatory effect and alleviates the neuroinflammation-mediated neurotoxicity induced by DJ-1 deficiency combined with LPS treatment. Many studies have also found that hyperactivation of the NF-κB pathway is closely associated with PD pathogenesis and is considered a promising intervention target for PD [44]. Increased nuclear translocation and transcriptional activity of NF-κB are observed in PD patients as well as in various PD models [41].

**Fig. 7:**
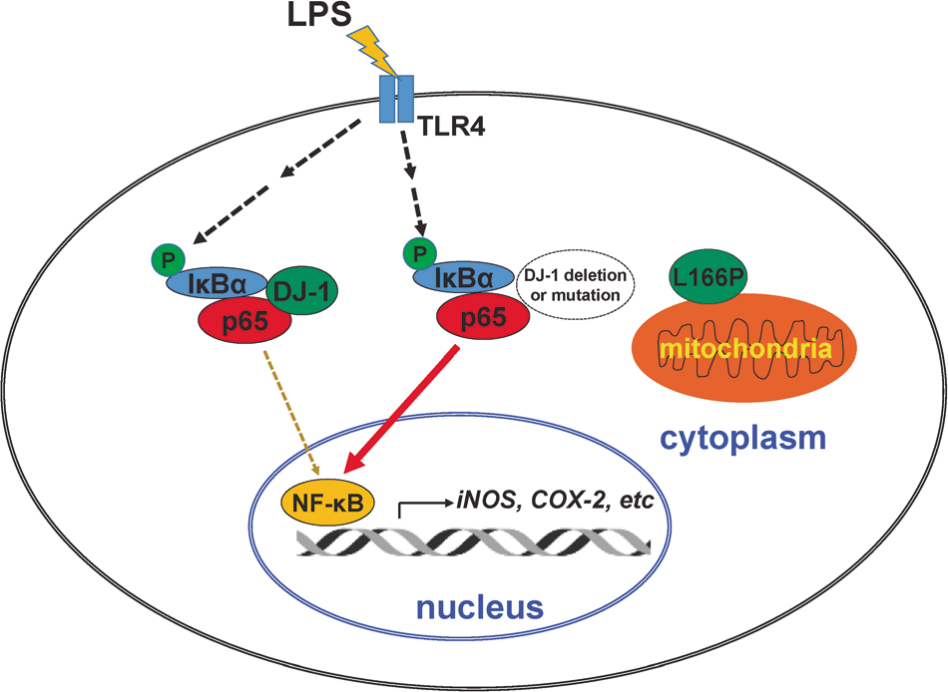
A schematic diagram shows that DJ-1 functions in neuroinflammation in microglia. Wild-type DJ-1 interacts with the subunit p65 of NF-κB and facilitates the interactions between IκBα and p65, thus weakens LPS-induced NF-κB nuclear transport and microglial activation. Whereas, loss of DJ-1 function by its deletion or L166P mutation which is mainly distributed in mitochondria and fails to bind to p65, augments LPS-induced microglial activation, and aggravates DA neuron damage.

In summary, we demonstrated that microglial wild-type DJ-1 but not DJ-1(L166P) represses neuroinflammation and neuroinflammation-mediated neurotoxicity by binding to the p65 subunit of NF-κB, and thus inhibiting NF-κB nuclear translocation and activation (Fig. 7). Loss of DJ-1 functions significantly increases microglia activation and dramatically aggregates microglia-mediated neurotoxicity in an NF-κB-dependent manner in response to LPS stimulation. In addition, inhibition of NF-κB nuclear translocation inhibits microglial activation and alleviates the loss of DA neurons induced by DJ-1 deficiency in vitro and in vivo.

## Materials and methods

### Animals

*DJ-1*^−/−^ mice were a kind gift from Dr. Jie Shen at Harvard Medical School [45]. The mice were kept in the SPF conditions. All mouse experiments were approved and carried out in accordance with the Regulations of Experimental Animal Administration, which are issued by the Animal Ethics Committee of Soochow University. For drug microfusion in vivo, 6- to 7-week-old mice were anesthetized and then fixed on the stereotaxic apparatus (RWD Life Science, Shenzhen, China). SN-50 (Selleck) was dissolved in ddH_2_O at a concentration of 0.1 μg/μl and loaded into a 5-μl Hamilton syringe fixed on the stereotaxic apparatus. SN-50 was microinjected into the SN (ML ± 1.2 mm, AP −3.3 mm, DV −4.6 mm) at the rate of 0.2 μl/min (1 μL/mouse). LPS (Sigma) (2 mg/mL) was microinjected into the mouse by the same method (2 μl/mouse). Fourteen days after LPS treatment, the mice were perfused with 0.9% saline, followed by 4% paraformaldehyde. Then, the brains of mice were removed and post-fixed overnight, followed by incubation with 30% sucrose solution overnight at 4 °C. Serial 20-µM-thick slices of midbrain were cut with a frozen microtome for immunohistochemistry. TH^+^-positive neurons in SN were counted for each slice under the microscope at a 10x magnification. The fluorescence intensity of IBA1 was analyzed using Photoshop7.0 software (Adobe, CA, USA).

### Primary microglia, cell line culture, and transfection

The procedure for extracting primary microglia from the newborn mouse cortex has been previously described [46]. The mouse microglial BV2 cells [47], mouse neuroblastoma Neuro2a (N2a) cells, and HEK293 cells were cultured in a DMEM medium (Gibco) with 10% fetal bovine serum, penicillin (100 U/ml), and streptomycin (100 µg/ml). All of the cell lines are authenticated by STR profiling. The small interfering RNAs (siRNAs) and the expression plasmids were transfected into cells with Lipofectamine RNAiMAX reagent (Invitrogen) and Lipofectamine2000 reagent (Invitrogen), respectively.

### Immunohistochemistry and immunocytochemistry

For immunohistochemistry, the slices of mouse midbrains were stained overnight with anti-IBA1 antibodies (019-19741, Wako, Japan) for labelling microglia, and anti-TH antibodies (AB152, Millipore) for DA neurons, and then incubated with the Alexa Fluor 488-conjugated fluorescent secondary antibody for 1 h. And anti-CD14 (60253-1-Ig, Proteintech Technology) antibody and the Alexa Fluor 594-conjugated secondary antibody (Invitrogen) were used for pro-inflammatory microglia staining. Then, the slices were labelled with 4′,6-diamidino-2-phenylindole (DAPI, Sigma) for 10 min to visualize cellular nuclei. For immunocytochemistry, fixed HEK293 cells were incubated with polyclonal anti-p65 (ab32536, Abcam) or anti-TOM20 (42406, Cell Signaling Technology) with monoclonal anti-FLAG (F3165, Sigma) antibodies for 4 h, and then incubated with Alexa Fluor 594- and 488-conjugated fluorescent secondary antibody for 2 h. For fixed BV2 cells, the primary polyclonal anti-p65 and Alexa Fluor 594-conjugated fluorescent secondary antibodies were applied, and then DAPI was used to stain the nuclei. Finally, the slices and cells were visualized using an IX71 inverted system microscope (Olympus, Japan), or an LSM800 confocal microscope (Zeiss, Germany).

### Plasmids, siRNAs, and drugs

The p3xFLAG-myc-cmv-24-DJ-1 and DJ-1(L166P), pGEX-5x-1-DJ-1 and DJ-1(L166P) plasmids have been previously described [19, 48]. The EGFP-p65 plasmid was a kind gift from Dr. Yizheng Wang. pET-15b-p65 has been previously described [49]. The pNF-κB-luc-containing quadruple NF-κB response element (GGGAATTTCC) was purchased from Beyotime Biotechnology Co. Ltd. (Nantong, China). Cignal lentiviral NF-κB-luc was purchased from Qiagen. The plasmid fidelities were identified by sequencing. siRNA sequences targeting mouse *DJ-1* mRNA were as follows: sense: 5′ -CGCUUGUUCUCAAAGACUATT-3′, anti-sense: 5′ -UAGUCUUUGAGAACAAGCGGT-3′.

### Immunoprecipitation assay and GST-pull-down assay

The procedures of immunoprecipitation assay using polyclonal anti-GFP antibodies (Roche), polyclonal anti-p65 antibodies (Cell Signaling Technology), and normal rabbit IgG (Santa Cruz Biotechnology) were according to our previous work [19]. Bound proteins and cell lysates (input) were analyzed by immunoblot using the indicated primary antibodies and the light chain-specific secondary antibodies (Jackson). For the pull-down assay, GST, GST-DJ-1, and GST-DJ-1(L166P) (20 μg) expressed in *Escherichia coli* strain JM109 were incubated with glutathione-Sepharose 4B (GE Healthcare) for 30 min in an ice bath, respectively. After washing three times, the beads were incubated with His-p65 (50 μg), which is expressed by *Escherichia coli* strain BL21 in an ice bath for 4 h. Subsequently, the beads were washed with cold PBS five times. The input representing 10% of the proteins used in the immunoprecipitation or pull-down assay and the interacting proteins were analyzed by immunoblotting.

### Immunoblotting and antibodies

Cells were dissolved in cell lysis buffer (0.5% deoxycholate, 1% NP-40, 50 mM Tris-HCl pH 7.5, 150 mM NaCl, and protease inhibitor cocktail (Roche)). The samples were separated by 10 or 15% SDS-PAGE and then transferred onto a polyvinylidene difluoride membrane (PVDF). The PVDF membranes were incubated with the primary antibodies as follows overnight at 4 °C: polyclonal anti-DJ-1 antibody (AB9212, Chemicon), monoclonal anti-GAPDH antibody (MAB374, Millipore), polyclonal anti-iNOS (ab15323), polyclonal anti-COX2 (ab15191), polyclonal anti-p65 (ab32536) and polyclonal anti-H2B (ab45695) antibodies (Abcam), polyclonal anti-IκBα antibody (4812, Cell Signaling Technology), monoclonal anti-FLAG antibody (F3165, Sigma), and monoclonal anti-DJ-1 (sc-55573) and anti-GFP (sc-9996) antibody (Santa Cruz). The secondary antibodies, consisting of horseradish peroxidase-conjugated sheep anti-mouse or anti-rabbit antibodies (Amersham Pharmacia Biotech), were used for visualization with enhanced chemiluminescence (ECL) detection kit (Amersham Biosciences) and the use of a Chemiluminescence Imaging System (Bioshine ChemiQ 4800).

### ELISA assay and NO measurement

The levels of PGE2 (Cayman Chemical Company), TNFα, and IL-6 (Boster Biological Technology) in 100 µl of BV2 cultured media were detected using enzyme-linked immunosorbent assay (ELISA) kits. The NO concentration was measured using the Griess method with an NO assay kit (Beyotime Biotechnology) according to the manufacturer’s instructions.

### Quantitative real-time PCR (qRT-PCR)

Total RNA was extracted from BV2 cells or primary microglia using TRIzol reagent (Invitrogen). Then, the RNA was reverse-transcribed into cDNA using PrimeScript RT Master Mix (Takara). Subsequently, quantitative measurement of the target mRNA was subjected to qRT-PCR with SYBR Green Real-Time PCR Master Mix within a 7500 real-time PCR system (Applied Biosystems). The following primers were used: mouse *COX-2*, 5′-CAGGCTGAACTTCGAAACA-3′ and 5′-GCTCACGAGGCCACTGATACCTA-3′; mouse *iNOS*, 5′-TCCCAGCCTGCCCCTTCAAT-3′ and 5′-CGGATCTCTCTCCTCCTGGG-3′; mouse *IL-6*, 5′-TAGTCCTTCCTACCCCAATTTCC-3′ and 5′-TTGGTCCTTAGCCACTCCTTC-3′; mouse *IL-1β*, 5′-GCAACTGTTCCTGAACTCAACT-3′ and 5′-ATCTTTTGGGGTCCGTCAACT-3′; and mouse *TNFα*, 5′-CCCTCACACTCAGATCATCTTCT-3′ and 5′-GCTACGACGTGGGCTACAG-3′; mouse *β-actin*, 5′-GACCTGACTGACTACCTC-3′ and 5′-GACAGCGAGGCCAGGATG-3′. The relative mRNA levels of these genes to *β-actin* were calculated by the 2^*−*ΔΔCT^ method.

### Nuclear and cytoplasmic fractionation assay

The procedure for extracting nuclear and cytoplasmic fractionation has been previously described [50]. Briefly, the BV2 cells were collected and dissolved in fractionation buffer (2 mM MgAc, 3 mM CaCl_2_, 320 mM sucrose, 1 mM dithiothreitol (DTT), 0.1 mM EDTA, 0.5% NP-40, and 0.5 mM phenylmethylsulfonyl fluoride (PMSF)) on ice for 20 min. After centrifugation at 600 x *g* at 4 °C for 15 min, the collected supernatant was the cytoplasmic fraction. The collected pellet was washed once using fractionation buffer without NP-40, and then dissolved in nuclear buffer (25% glycerol, 20 mM HEPES (pH 7.9), 1.5 mM MgCl_2_, 0.2 mM EDTA, 280 mM KCl, 1 mM DTT, 0.3% NP-40, and 0.5 mM PMSF) as the nuclear fraction.

### Luciferase reporter gene assay

BV2 cells stably expressing Cignal lentiviral NF-κB-luc were constructed by limited dilution method and selected with puromycin (2.5 μg/ml) (Invitrogen) following transfection. BV2 cells stably expressing Cignal lentiviral NF-κB-luc were transfected with si-*Ctrl* or si-*DJ-1*. After 48 h, the cells were treated with 100 ng/ml of LPS or the equal volume PBS for 24 h. In HEK293 cells, cells were transfected with FLAG-DJ-1, FLAG-DJ-1(L166P), or empty vector, along with pNF-κB-luc and the *Renilla* luciferase vector pRL-CMV, which acts as a normalized control. After 48 h, firefly and *Renilla* luciferase activities were detected with a dual-luciferase reporter system (Promega) using a microplate reader (Infinite M1000 Pro, Tecan).

### Conditioned medium assays

The procedure for conditioned medium assays has been previously described [51]. Briefly, si-*Ctrl* or si-*DJ-1* were transfected into BV2 cells for 72 h, and then cells were pretreated with or without SN-50 (20 μM) for 1 h, followed by treatment with or without LPS (100 ng/ml) (Sigma) for 24 h. The cells were then washed twice with PBS and cultured for another 24 h in fresh media to generate the conditioned media. The conditioned media of BV2 cells was then collected and filtered by a 0.22 μm filter to culture N2a cells for 24 h. The N2a cells were then stained with 1 µM PI (Sigma) and visualized with an inverted microscope IX71 (Olympus, Japan). The PI-positive cells were counted and analyzed.

### Statistical analysis

The densitometric values of three independent immunoblotting experiments were calculated by Photoshop 7.0 software (Adobe). The data presented as the mean ± SD from at least three independent experiments were analyzed by GraphPad Prism 8.0 software (San Diego, CA, USA). The statistical significance of the differences between groups was determined using *t*-test (two-tailed) following two-way ANOVA unless otherwise specified in the figure legends. The criterion of significance was set at *P* < 0.05. In this study, *P* values less than 0.001 were shown as ***P < 0.001, *P* values less than 0.01 were shown as **P < 0.01, *P* values less than 0.05 were shown as *P < 0.05, and no statistical significance was shown as “ns”. The sample size of experiments is determined on basis of literature in this field. No sample was excluded from the analysis. Animals were carefully assigned based on genotype and age, rather than randomly. There were no studies in which investigators were blinded. The replicate numbers are described in each figure legend.
